# Developmental Programming of Adult Disease: Reprogramming by Melatonin?

**DOI:** 10.3390/ijms18020426

**Published:** 2017-02-16

**Authors:** You-Lin Tain, Li-Tung Huang, Chien-Ning Hsu

**Affiliations:** 1 Department of Pediatrics, Kaohsiung Chang Gung Memorial Hospital and Chang Gung University College of Medicine, Kaohsiung 833, Taiwan; tainyl@hotmail.com (Y.-L.T.); litung.huang@gmail.com (L.-T.H.); 2 Institute for Translational Research in Biomedicine, Kaohsiung Chang Gung Memorial Hospital and Chang Gung University College of Medicine, Kaohsiung 833, Taiwan; 3 Department of Traditional Chinese Medicine, Chang Gung University, Linkow 244, Taiwan; 4 Department of Pharmacy, Kaohsiung Chang Gung Memorial Hospital, Kaohsiung 833, Taiwan; chien_ning_hsu@hotmail.com; 5 School of Pharmacy, Kaohsiung Medical University, Kaohsiung 807, Taiwan

**Keywords:** cardiovascular disease, developmental origins of health and disease (DOHaD), developmental programming, epigenetic regulation, glucocorticoid, hypertension, melatonin, next-generation sequencing, non-communicable disease, oxidative stress, renin-angiotensin system

## Abstract

Adult-onset chronic non-communicable diseases (NCDs) can originate from early life through so-called the “developmental origins of health and disease” (DOHaD) or “developmental programming”. The DOHaD concept offers the “reprogramming” strategy to shift the treatment from adulthood to early life, before clinical disease is apparent. Melatonin, an endogenous indoleamine produced by the pineal gland, has pleiotropic bioactivities those are beneficial in a variety of human diseases. Emerging evidence support that melatonin is closely inter-related to other proposed mechanisms contributing to the developmental programming of a variety of chronic NCDs. Recent animal studies have begun to unravel the multifunctional roles of melatonin in many experimental models of developmental programming. Even though some progress has been made in research on melatonin as a reprogramming strategy to prevent DOHaD-related NCDs, future human studies should aim at filling the translational gap between animal models and clinical trials. Here, we review several key themes on the reprogramming effects of melatonin in DOHaD research. We have particularly focused on the following areas: mechanisms of developmental programming; the interrelationship between melatonin and mechanisms underlying developmental programming; pathophysiological roles of melatonin in pregnancy and fetal development; and insight provided by animal models to support melatonin as a reprogramming therapy. Rates of NCDs are increasing faster than anticipated all over the world. Hence, there is an urgent need to understand reprogramming mechanisms of melatonin and to translate experimental research into clinical practice for halting a growing list of DOHaD-related NCDs.

## 1. Introduction

Chronic non-communicable diseases (NCDs) are the leading cause of death in the world [1]. In spite of recent medical advances, the burden of NCDs is still rising globally [1]. NCDs can begin in early life and that brings out a concept, namely the “developmental origins of health and disease” (DOHaD) or “developmental programming” [2]. The plasticity of the developmental process allows the organism to adapt to changing environments to improve its odds of survival. Developmental programming is defined as the process by which an insult applied at a developmental window causes long-term effects on the structure or function of an organism [3]. The concept of developmental programming also affords the “reprogramming” strategy to shift the therapeutic approach from adulthood to early life, before clinical disease is evident [4].

Melatonin, an endogenous indoleamine secreted by the pineal gland, has pleiotropic bioactivities those are involved in circadian rhythm, reproductive physiology, anti-inflammation, redox homeostasis, epigenetic regulation, and fetal development [5,6,7,8,9,10]. A growing body of clinical and experimental studies supports that early-life suboptimal environment programs later susceptibility to certain chronic diseases throughout the life course. On the other hand, melatonin has emerged as a common reprogramming strategy to prevent a variety of diseases in different models of developmental programming. Here, we review the reported impacts of melatonin on developmental programming. We have particularly focused on the following areas: mechanisms of developmental programming, pathophysiological role of melatonin in pregnancy and fetal development; and current evidence of melatonin as a reprogramming therapy in models of developmental programming.

## 2. Mechanisms of Developmental Programming

### 2.1. Insight Provided by Human Studies

Several lines of evidence indicate that there is an association between suboptimal fetal and neonatal environments and development of adult disease in later life. One of the important observations from the Dutch Hunger Winter Study was that malnutrition during gestation has long-lasting consequences for adult health [11]. Offspring exposed to the famine during pregnancy are prone to develop a variety of adult diseases such as type 2 diabetes, coronary artery disease, obesity, dyslipidemia, and hypertension [11]. Another line of evidence supporting DOHaD concept comes from mother-child cohort studies. As reviewed elsewhere [4], several early-life risks related to developmental programming of hypertension has been assessed in various mother-child cohorts, including undernutrition, smoking, gestational hypertension, maternal obesity, short-term breastfeeding, low vitamin D intake, and excessive postnatal weight gain. Third are studies of twins. Studies of monozygotic and dizygotic twins provide a natural study design to assess the relative contribution of heretical and environmental factors on the developmental programming of adult diseases, such as cardiovascular disease (CVD) [12]. In twins, there was an association between birth weight and blood pressure (BP) in infants [13]. Additionally, the lighter twins are prone to die from heart disease in later lifespan [13].

However, these clinical studies cannot per se directly establish a causal relationship between the specific insult and phenotypes of programmed disease. A few DOHaD theories have been established to explain these epidemiological observations between early life attributes and later chronic diseases, such as thrifty phenotype [14], predictive adaptive responses [15], and catch-up growth hypothesis [16]. However, these hypotheses do not suggest possible molecular mechanisms by which way the phenotype is generated. It stands for reason that much of our knowledge of which types of early-life insults, which developmental window is critical for programming, and which one reprogramming strategy can be used mainly come from studies in animal models.

### 2.2. Insight Provided by Animal Models

The four main types of NCDs are CVD, cancers, chronic respiratory diseases, and diabetes [1]. In the past decades, a great number of animal models have been established to study developmental programming. Most of the long-term health outcomes in offspring often include insulin resistance, obesity, CVD, and metabolic syndrome, whatever the studied species [4,17,18,19,20,21]. Evidence is now emerging that, in addition to CVD and diabetes, developmental reprogramming can also influence the risk of developing cancer [22] as well as chronic lung disease [23].

To date, no unifying origin that can explain the pathogenesis of NCDs has been identified and, therefore, no specific reprogramming strategy is existing to avoid developmental programming of NCDs. Nevertheless, a number of mechanisms, including oxidative stress, epigenetic regulation, glucocorticoid effect, and alterations of renin-angiotensin system (RAS) have been reported to be associated with developmental programming of various adult diseases and different early-life insults [3,4,24,25,26,27]. Importantly, melatonin seems closely inter-related to each proposed mechanism as a hub in determining the programmed process.

### 2.3 The Interrelationship between Melatonin and Underlying Mechanisms of Developmental Programming

**Figure 1:**
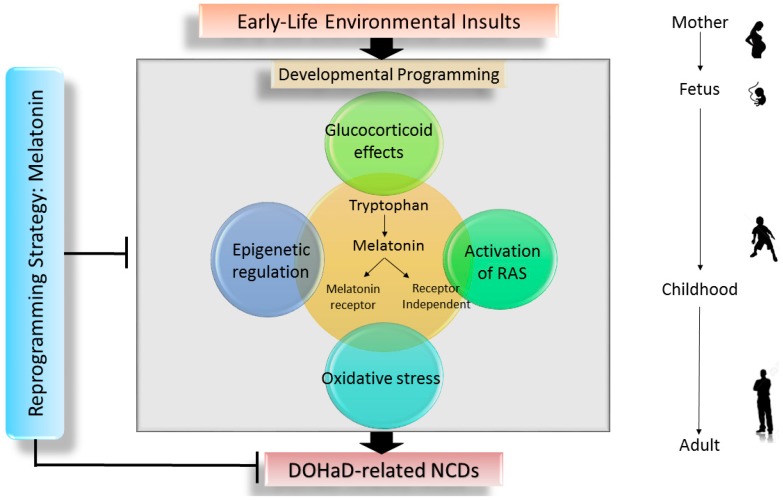
Schema outlining the early-life insults that drive the potential mechanisms that may underpin the developmental programming, leading to developmental origins of health and disease (DOHaD)-related non-communicable diseases (NCDs) in adulthood. There is a link between melatonin synthesis and signaling pathway close to oxidative stress, epigenetic regulation, glucocorticoid effect, and renin-angiotensin system (RAS). Early intervention with melatonin could be a reprogramming strategy to prevent the development of DOHaD-related NCDs in later life.

Currently, more and more people are turning to be active during night time (e.g., shift work and jet lag), resulting in reduced nocturnal melatonin levels and circadian disruption. It is noteworthy that circadian disruption can increase the risk of CVD, diabetes, cancer, and metabolic syndrome [28,29]. In contrast, melatonin, a chronobiotic hormone, has been considered as a chronotherapeutic drug for these NCDs [28]. Additionally, several lines of evidence indicate that melatonin interacts with these proposed mechanisms contributing to programmed process. First, convincing evidence indicate that melatonin plays a protective role against the oxidative stress [6,10,30]. Oxidative stress is a result of damage to radicals and related molecules, in particular reactive oxygen species (ROS) and reactive nitrogen species (RNS). As known, melatonin is a major scavenger of both oxygen and nitrogen-based reactive molecules [30]. The main sources of nitric oxide (NO) are the reactions catalyzed by neuronal, inducible, and endothelial NO synthase (nNOS, iNOS, and eNOS). Melatonin has been reported to inhibit nNOS [31] and iNOS [32], to curtail the generation of the highly toxic nitrogen-based reactant, ONOO^-^. On the other hand, melatonin seems to increase eNOS-derived NO bioavailability with subsequent vasodilatation via enhancement of intracellular Ca^2+^ level [33]. Additionally, melatonin and its metabolites are potent antioxidants [10]. Moreover, melatonin can be protective as a reprogramming intervention to restore the NO-ROS balance in both genetic and developmentally programmed hypertension models [4]. Second are many reports that melatonin has epigenetic property [5,9,33]. Epigenetics refers to alterations in gene expression that are not explained by changes in DNA sequence. DNA methylation, histone modification and RNA interference play central roles in epigenetic regulation [34]. Previous reports showed that melatonin can inhibit DNA methyltransferases (DNMT) or act like a histone deacetylase (HDAC) inhibitor [5,33]. Given that epigenetic changes due to early-life insults predispose the offspring to develop a variety of diseases later in life and, thus, melatonin, may act as an epigenetic regulator to reprogram the process and prevent the development of adult disease. Third are studies of crosstalk between glucocorticoid and melatonin [35]. Melatonin receptor (MT) expression has been reported to be downregulated following dexamethasone treatment [36], while glucocorticoid receptor (GR) expression can be downregulated in response to melatonin [35]. Additionally, our previous studies demonstrated that melatonin therapy is protective on prenatal dexamethasone-induced programming of steatosis [37], cognition deficit [38], and hypertension [36,39,40]. Therefore, these findings suggest that a pathophysiological cross talk between glucocorticoid and melatonin which is of significance in developmental programming. Last, in melatonin-deficient hypertension model, the RAS is activated [41]. In contrast, melatonin treatment can regulate RAS components and prevent the elevation of BP in several programming models of hypertension [35,40,42,43]. All of these observations provide a close link between melatonin and other mechanisms involved in developmental programming (Figure 1). Thus, early intervention with melatonin presumably could be a reprogramming strategy to prevent the development of NCDs in later life.

## 3. The Impact of Melatonin on Pregnancy and Fetal Development

### 3.1. Synthesis, Metabolism, and Signaling Pathway of Melatonin

Melatonin (*N*-acetyl-5-methoxytryptamine) is an endogenously produced indoleamine containing two functional groups, which are for the receptor binding and capacity to enter cells. Tryptophan is a precursor for melatonin biosynthesis. At least four enzymes are involved in the synthesis of melatonin. Among them, serotonin *N*-acetyltransferase is considered the rate-limiting enzyme in the regulation of melatonin biosynthesis. Once released into the blood, 70% of melatonin is bound to albumin, and another 30% diffuses to the surrounding tissues [44]. Circulating melatonin is mainly secreted during the night by the pineal gland, while almost all organs can produce melatonin [44]. Melatonin is mainly catabolized by the hepatic P450 monooxygenase, followed by conjugation of the resulting 6-sulfatoxy-melatonin to give the main urinary metabolite 6-sulftory-melatonin. Melatonin can interact with two transmembrane melatonin receptors, melatonin receptor-1 (MT1) and -2 (MT2), as well as retinoid related orphan nuclear hormone receptors of the RZR/ROR family for signal transduction [10,44]. Melatonin has multiple receptor-dependent and receptor-independent functions, such as antioxidant and anti-inflammatory properties, free radical scavenger, regulation of circadian rhythm, stimulatory action in the immune system and mitochondrial biogenesis, and epigenetic regulation [5,6,7,8,9,10,44]. Moreover, melatonin is important in pregnancy, parturition, and fetal development [7,8,44,45,46,47,48].

### 3.2. Melatonin in Pregnancy and Fetus

Melatonin plays a crucial role in pregnancy and fetal development [45,46,47,48]. In normal pregnancy, melatonin acts as a circadian rhythm modulator, free radical scavenger, antioxidant, and immunomodulator, leading to successful pregnancy [45]. Plasma melatonin levels are elevated during pregnancy, reaching a maximum at term, and returning to basal levels immediately after delivery [46]. Placentas not only produce melatonin but also express melatonin receptors and clock genes [46]. Thus, melatonin deficiency in pregnancy links circadian disruption with compromised placental function [47]. In preeclamptic placentas, the expression of melatonin-synthesizing enzymes is reduced, combined with decreased melatonin levels and melatonin receptor expression [48]. Moreover, maternal melatonin can cross the placenta and enter the fetal circulation to drive the fetal circadian system.

In rodents, melatonin-binding sites are observed in the fetal pituitary gland as early as gestational age of 15 days [49]. Melatonin receptors are extensive expression in the embryo and fetus since early stages. Therefore, maternal melatonin may be involved in early stage of fetal development. The maternal melatonin circadian rhythm is linked to the generation of the circadian rhythms in fetus [50]. While genetic disruption of maternal and embryonic clock function results in abnormal organogenesis in fetus [51]. Administration of melatonin was reported to restore redox balance to alleviate maternal hyperthermia-induced embryo death [52]. Additionally, melatonin prevented preterm labor and increased offspring survival in a mouse model of lipopolysaccharide (LPS)-induced inflammation [53]. In a continuous light exposure-induced melatonin deficiency pregnant rat model, offspring developed intrauterine growth retardation and disrupted circadian, which were prevented by maternal melatonin treatment [54]. Likewise, maternal melatonin regulates fetal organogenesis that are critical for the successful adaptation of the neonate to extra-uterine environment [55]. Accordingly, these observations indicate that maternal melatonin plays a vital role to prevent pregnancy loss and preserve normal fetal development.

## 4. Melatonin as a Reprogramming Therapy in Animal Models of Developmental Programming

Emerging evidence has shown multiple protective actions of melatonin on many human diseases across all age groups [7,10,56,57,58,59,60,61,62]. Since melatonin controls circadian rhythm, several clinical trials were focused on its therapeutic effects for sleep disorders and related neurological diseases [60]. Additionally, the therapeutic roles of melatonin in human CVDs, such as coronary artery disorder, hypertension, congestive heart failure, and pulmonary hypertension are gradually being recognized [59,61]. Furthermore, the clinical impact of melatonin has been evaluated in cancer therapy [58,62], in the immune function [58], in obesity and diabetes [62], and in disorders related to oxidative stress [10]. The list of clinical conditions in which melatonin therapy is beneficial continues to grow. However, less attention has been paid to examine melatonin as a reprogramming therapy in the research field of DOHaD. The overview of studies in Table 1 illustrates data documenting reprogramming effects of melatonin treatment in different developmental programming animal models. As shown in Table 1, a variety of early-life insults have been reported to cause developmental programming of adult diseases. These environmental and nutritional insults include maternal undernutrition [42], *N*^G^-nitro-l-arginine-methyester (L-NAME) induced preeclampsia [43], high-fructose consumption [63], prenatal hypoxia [64], glucocorticoid exposure [33,36,37,38,39,40,65], high-fat diet [40], and constant light exposure [66]. These insults induce a number of programming effects on adult offspring including hypertension, reduced nephron number, cognition deficit, behavior dysfunction, obesity, and liver steatosis. All these programmed phenotypes can be prevented, or at least alleviated, by early melatonin administration. It is noteworthy that melatonin in these models of developmental programming is mainly administered during pregnancy and lactation, that is, during the developmental stage before clinical disease is evident. Thus, the influence of melatonin on adult offspring should be considered as reprogramming instead of direct effects. So far, a few mechanisms, including oxidative stress [42,43,63,65], epigenetic regulation [38,39,40], reduction in nephron numbers [39], and alterations of the RAS [40] have been linked to the reprogramming effects of melatonin.

Despite the protective role of early melatonin treatment has been assessed in a number of models of developmental programming, the knowledge of mechanisms driving reprogramming effects, appropriate windows for reprogramming intervention, and ideal dose and timing are necessary to provide melatonin as a reprogramming strategy to halt the rise in NCDs in future research efforts.

## 5. Long-Term Effects of Melatonin in Normal Offspring

In humans, melatonin therapy has a remarkably benign safety profile across different populations [67]. Although melatonin has been reported to influence body weight in some human and experimental studies [39,68], a recent meta-analysis of 244 cases from seven trials did not provide much support [69]. So far, only pregnant and breast-feeding women are not recommended for melatonin use due to lack of human studies [67]. In pregnant rats, melatonin has shown no harmful effects on the development of rat pups, even at high doses up to 200 mg/kg/day [70,71]. However, our recent reports suggest that maternal melatonin therapy has long-term profound effects on transcriptomic changes in specific organs of fetal programming [9,40,42,63]. Although melatonin has been shown to elicit a panel of gene expression in different organs and diseases [72,73], only few studies reported its long-term programming effects on organ transcriptome of normal offspring [9,40].

We used to employ whole-genome RNA next-generation sequencing (NGS) to analyze renal transcriptome from three different ages of male offspring born to mothers exposed to 0.01% melatonin in drinking water during pregnancy and lactation [9]. We observed 455, 230, and 132 differentially expressed genes were noted in response to maternal melatonin administration in offspring at 1-, 12-, and 16-week of age, respectively. In agreement with our other reports [40,42,63], maternal melatonin therapy is likely to up-regulate, but not down-regulate, genes in the offspring kidney. Since previous studies show that melatonin acts like a DNMT or HDAC inhibitor [5,33], our findings support melatonin may serve as an inducer of gene expression in the offspring kidney. Additionally, we found melatonin can up-regulate several epigenetic regulator genes during nephrogenesis. However, melatonin-mediated transcriptomic changes may decline in a time-dependent manner [9].

Given that melatonin regulates many physiological functions, it is not surprising that our NGS data demonstrated numerous biological pathways are regulated by melatonin administration during nephrogenesis [40]. Using DAVID v6.8 bioinformatics tool [74], there were 20 significantly related Kyoto Encyclopedia of Genes and Genomes (KEGG) pathways being identified in the offspring kidney (Table 2). It is noteworthy that the tryptophan metabolism pathway is regulated by maternal melatonin administration as tryptophan is a precursor of melatonin synthesis. Indeed, we observed that genes involved in the biosynthetic pathway of melatonin were significantly up-regulated, including *Tph1* (encoded for tryptophan hydroxylase 1), *Ddc* (encoded for aromatic l-amino acid decarboxylase), and *Asmt* (encoded for *N*-acetylserotonin methyltransferase). Despite *Mtnr1a* (encoded for MT1) was undetectable, other melatonin receptors *Mtnr1b*, *Rora*, and *Rorb* were up-regulated in the offspring kidney born to mothers exposed to melatonin treatment. These findings indicate that that maternal melatonin therapy can program the synthesis, metabolism, and signaling pathway of melatonin in the developing offspring kidney [40].

Whether these programmed processes and pathways might be protective mechanisms of melatonin to prevent a number of programmed diseases remain to be elucidated. Despite our NGS data indicate that transcriptomic changes associated with programming by early melatonin therapy may be lessened in later life, early melatonin therapy might cause long-term transcriptomic changes to what extent, however, deserves to be elucidated.

## 6. Conclusions

The global burden of NCDs continues to increase, despite advances in medical, surgical, and critical care. Since suboptimal environments leading to developmental programming are often multifactorial, identification of common reprogramming strategies from animal models become a practical approach for further translation to the clinic, to halt the global burden of NCDs. This review has summarized a broad spectrum of models of developmental programming relevant to the reprogramming effects of melatonin in the literature, but it is still not complete. Although some progress has been made in research on melatonin as a reprogramming strategy in animal studies, but many challenges still lie ahead. What are missing from the literature include deeper understandings of how melatonin drives reprogramming effects, which developmental window is appropriate for reprogramming, and which doses and timing are ideal for reprogramming are essential to developing melatonin as a reprogramming therapy to treat a growing list of diseases of DOHaD-related NCDs.

## Figures and Tables

**Table 1:** Effects of melatonin on developmental programming in various animal models.

Programming Models	Melatonin Treatment	Reprogramming Effects	Age at Evaluation	Ref.
50% caloric restriction during pregnancy and lactation	0.01% melatonin in drinking water during pregnancy and lactation	Prevented hypertension, Increased renal NO	3 mo	[42]
l-NAME 60 mg/kg/day subcutaneously during pregnancy	0.01% melatonin in drinking water during pregnancy and lactation	Prevented hypertension, Increased renal NO	3 mo	[43]
60% high fructose intake during pregnancy and lactation	0.01% melatonin in drinking water during pregnancy and lactation	Prevented hypertension, Increased renal NO	3 mo	[63]
Phenytoin 50 mg/kg orally from gestational day 7 to 18	Melatonin (40 μg/mL) in drinking water from gestational day 0 to 19	Protected neurobehavioral dysfunctions	3 mo	[64]
Neonatal dexamethasone exposure	0.01% melatonin in drinking water during pregnancy and lactation	Prevented hypertension, Preserved histone deacetylase gene expression	4 mo	[33]
Neonatal dexamethasone exposure	0.01% melatonin in drinking water during lactation	Prevented hypertension, Preserved renal melatonin receptor-2 protein, Increased renal melatonin level	4 mo	[36]
Prenatal dexamethasone exposure	0.01% melatonin in drinking water during pregnancy and lactation	Reversed methylation of leptin, Decreased liver steatosis	4 mo	[37]
Prenatal dexamethasone exposure	0.01% melatonin in drinking water during pregnancy and lactation	Reversed hippocampal morphology and reelin level	4 mo	[38]
Prenatal dexamethasone exposure	0.01% melatonin in drinking water during pregnancy and lactation	Prevented hypertension, Increased nephron number	4 mo	[39]
Prenatal dexamethasone exposure plus post-weaning high-fat diet	0.01% melatonin in drinking water during pregnancy and lactation	Prevented hypertension, Upregulated *Agtr1b* and *Mas1* expression	4 mo	[40]
Corticosterone 1 µg/day in the morning from postnatal day 2 to 14	Melatonin 40 μg/day at night from postnatal day 2 to 14	Protected diabetic manifestations and oxidative stress	4 mo	[65]
Constant light exposure from gestational day 10 to 21	Melatonin 1 mg/kg at circadian time 12, from day 17 to 21 of pregnancy	Protected anxiety-like and sexual behaviors	4 mo	[66]

Studies tabulated according to age at evaluation. mo = month.

**Table 2:** Significantly regulated Kyoto Encyclopedia of Genes and Genomes (KEGG) pathways in the one-week-old offspring kidney born to mothers treated with melatonin versus control.

Term	Count	%	*p*-Value	Benjamini
Focal adhesion	50	2.3	4.4 × 10^−8^	1.2 × 10^−5^
Regulation of actin cytoskeleton	49	2.3	3.9 × 10^−7^	5.6 × 10^−5^
Pathways in cancer	75	3.5	4.8 × 10^−7^	4.5 × 10^−5^
Axon guidance	32	1.5	6.0 × 10^−6^	4.3 × 10^−4^
*ErbB* signaling pathway	25	1.2	1.5 × 10^−5^	8.3 × 10^−4^
AMPK signaling pathway	30	1.4	3.9 × 10^−5^	1.9 × 10^−3^
Metabolic pathways	177	8.2	6.7 × 10^−5^	2.7 × 10^−3^
Chemokine signaling pathway	37	1.7	6.8 × 10^−5^	2.4 × 10^−3^
Insulin signaling pathway	31	1.4	1.0 × 10^−4^	3.2 × 10^−3^
Proteoglycans in cancer	40	1.8	1.0 × 10^−4^	2.9 × 10^−3^
PI3K-Akt signaling pathway	59	2.7	1.1 × 10^−4^	2.7 × 10^−3^
Ras signaling pathway	44	2.0	1.2 × 10^−4^	2.9 × 10^−3^
Adherens junction	20	0.9	1.4 × 10^−4^	3.0 × 10^−3^
PPAR signaling pathway	20	0.9	2.9 × 10^−4^	5.9 × 10^−3^
Butanoate metabolism	11	0.5	3.5 × 10^−4^	6.6 × 10^−3^
Renal cell carcinoma	18	0.8	4.2 × 10^−4^	7.4 × 10^−3^
MAPK signaling pathway	46	2.1	4.4 × 10^−4^	7.3 × 10^−3^
mTOR signaling pathway	17	0.8	5.0 × 10^−4^	7.9 × 10^−3^
Prostate cancer	21	1.0	6.4 × 10^−4^	9.6 × 10^−3^
Tryptophan metabolism	14	0.6	6.5 × 10^−4^	9.3 × 10^−3^

## Abbreviations

CVD	Cardiovascular disease
DNMT	DNA methyltransferases
DOHaD	Developmental origins of health and disease
HDAC	Histone deacetylase
KEGG	Kyoto Encyclopedia of Genes and Genomes
l-NAME	*N*^G^-nitro-l-arginine-methyester
MT	Melatonin receptor
NCD	Non-communicable disease
NGS	Next generation RNA sequencing
NOS	Nitric oxide synthase
RAS	Renin-angiotensin system
ROS	Reactive oxygen species
